# SARS-CoV-2 B.1.1.529 (Omicron) Variant Transmission Within Households — Four U.S. Jurisdictions, November 2021–February 2022

**DOI:** 10.15585/mmwr.mm7109e1

**Published:** 2022-03-04

**Authors:** Julia M. Baker, Jasmine Y. Nakayama, Michelle O’Hegarty, Andrea McGowan, Richard A. Teran, Stephen M. Bart, Katie Mosack, Nicole Roberts, Brooke Campos, Alina Paegle, John McGee, Robert Herrera, Kayla English, Carla Barrios, Alexandria Davis, Christine Roloff, Lynn E. Sosa, Jessica Brockmeyer, Lindsey Page, Amy Bauer, Joshua J. Weiner, Manjeet Khubbar, Sanjib Bhattacharyya, Hannah L. Kirking, Jacqueline E. Tate

**Affiliations:** ^1^CDC COVID-19 Emergency Response Team; ^2^Epidemic Intelligence Service, CDC; ^3^Oak Ridge Institute for Science and Education, Oak Ridge, Tennessee; ^4^Chicago Department of Public Health; ^5^Connecticut Department of Public Health; ^6^Milwaukee Health Department; ^7^Utah Department of Health; ^8^CDC Foundation, Atlanta, Georgia.

The B.1.1.529 (Omicron) variant, first detected in November 2021, was responsible for a surge in U.S. infections with SARS-CoV-2, the virus that causes COVID-19, during December 2021–January 2022 (*1*). To investigate the effectiveness of prevention strategies in household settings, CDC partnered with four U.S. jurisdictions to describe Omicron household transmission during November 2021–February 2022. Persons with sequence-confirmed Omicron infection and their household contacts were interviewed. Omicron transmission occurred in 124 (67.8%) of 183 households. Among 431 household contacts, 227 were classified as having a case of COVID-19 (attack rate [AR] = 52.7%).^†^ The ARs among household contacts of index patients who had received a COVID-19 booster dose, of fully vaccinated index patients who completed their COVID-19 primary series within the previous 5 months, and of unvaccinated index patients were 42.7% (47 of 110), 43.6% (17 of 39), and 63.9% (69 of 108), respectively. The AR was lower among household contacts of index patients who isolated (41.2%, 99 of 240) compared with those of index patients who did not isolate (67.5%, 112 of 166) (p-value <0.01). Similarly, the AR was lower among household contacts of index patients who ever wore a mask at home during their potentially infectious period (39.5%, 88 of 223) compared with those of index patients who never wore a mask at home (68.9%, 124 of 180) (p-value <0.01). Multicomponent COVID-19 prevention strategies, including up-to-date vaccination, isolation of infected persons, and mask use at home, are critical to reducing Omicron transmission in household settings.

Persons with sequence-confirmed Omicron variant infections during November 2021–February 2022 were identified from four U.S. jurisdictions (Chicago, Illinois; Connecticut; Milwaukee, Wisconsin; and Utah) and contacted by telephone to assess eligibility of the household to participate in the investigation.^§^ A household was eligible if the index patient did not live in a congregate setting and did live with at least one other person for most of their potentially infectious period, defined as 2 days before through 10 days after the index date (the date of the index patient’s positive SARS-CoV-2 nucleic acid amplification test result or antigen test result or symptom onset, whichever occurred first). Index patients were defined as the first person within each household to recently experience COVID-19–compatible symptoms^¶^ or have a positive SARS-CoV-2 test result. Household contacts were defined as any persons who spent one or more overnights in the residence with the index patient during their potentially infectious period. If it was unclear who within the household was the index patient (e.g., if multiple persons developed COVID-19–compatible symptoms in the household on the same day or had the same SARS-CoV-2 exposure) or if household contacts had confirmed or probable cases and were known to have a SARS-CoV-2 exposure to someone other than the index patient, the household was excluded from analyses.

Index patients and household contacts participated in voluntary telephone interviews to retrospectively collect information on demographic characteristics, SARS-CoV-2 testing, symptoms, COVID-19 vaccination history, previous SARS-CoV-2 infection, index patient isolation practices (defined as always or sometimes isolating in a room by oneself at any point during their potentially infectious period), and index patient mask use practices (defined as ever wearing a mask at home during their potentially infectious period). For this investigation, a confirmed case in a household contact was defined as a positive SARS-CoV-2 nucleic acid amplification test result or antigen test result (through local or home testing)** ≤14 days after the index date. A probable case in a household contact was defined as the presence of COVID-19–compatible symptoms in a household contact during the same 14-day period, but without confirmation by a SARS-CoV-2 test.^††^ Vaccination status was based primarily on self-report^§§^; participants were categorized as having received a booster dose, fully vaccinated (<5 or ≥5 months before the index date), partially vaccinated, or unvaccinated.^¶¶^

The interval between the index date and onset of symptoms or positive test result in a household contact was calculated. ARs among household contacts were estimated overall, by household contact characteristics, and by index patient characteristics, by dividing the number of household contacts with confirmed and probable cases by the total number of household contacts within a given stratum. P-values comparing differences in stratum-specific ARs were calculated using a generalized estimating equation approach to account for clustering by household (*2*). Statistical significance was defined as p<0.05. Subanalyses were conducted to examine potential secondary transmission (as opposed to all household transmission); the interval was calculated for households of two persons (index patient and another household contact), and ARs were calculated after restricting the case definition to cases identified ≤7 days*** after the index date. Data were collected and managed using REDCap (version 11.1.8; Vanderbilt University) and analyzed using R (version 4.0.3; R Foundation). This activity was reviewed by CDC and was conducted consistent with applicable federal law and CDC policy.^†††^

A total of 3,558 persons were considered potentially eligible for participation in the investigation, among whom jurisdictions attempted to contact 1,461 (41.1%). Of the 562 households successfully contacted, 175 (31.1%) declined to participate, and 204 (36.3%) were excluded; 183 (32.6%) were enrolled.^§§§^ Enrolled households included 183 index patients and 439 household contacts (Table). The median index patient age was 37 years (IQR = 23–54 years). A majority of index patients were White (59.0%, 108 of 183), and 21.3% (39 of 183) were Hispanic/Latino.

**TABLE:** Characteristics* and vaccination status of index COVID-19 patients (n = 183) and their household contacts (n = 439) — four U.S. jurisdictions, November 2021–February 2022

Characteristic	No. (column %)		
	Index patients, n = 183	Household contacts, n = 439	Total, N = 622
**Jurisdiction**			
Chicago, Illinois	26 (14.2)	51 (11.6)	**77 (12.4)**
Connecticut	93 (50.8)	218 (49.7)	**311 (50.0)**
Milwaukee, Wisconsin	36 (19.7)	101 (23.0)	**137 (22.0)**
Utah	28 (15.3)	69 (15.7)	**97 (15.6)**
**Age group, yrs^†^**			
0–4	8 (4.4)	41 (9.3)	**49 (7.9)**
5–11	11 (6.0)	51 (11.6)	**62 (10.0)**
12–17	14 (7.7)	42 (9.6)	**56 (9.0)**
18–64	134 (73.2)	262 (59.7)	**396 (63.7)**
≥65	14 (7.7)	27 (6.2)	**41 (6.6)**
Unknown	2 (1.1)	16 (3.6)	**18 (2.9)**
**Gender**			
Female	95 (51.9)	229 (52.2)	**324 (52.1)**
Male	88 (48.1)	199 (45.3)	**287 (46.1)**
Unknown	0 (—)	11 (2.5)	**11 (1.8)**
**Race**			
White	108 (59.0)	209 (47.6)	**317 (51.0)**
Black	27 (14.8)	35 (8.0)	**62 (10.0)**
Asian	15 (8.2)	25 (5.7)	**40 (6.4)**
Other/Multiple^§^	16 (8.7)	33 (7.5)	**49 (7.9)**
Unknown	17 (9.3)	137 (31.2)	**154 (24.8)**
**Ethnicity**			
Non-Hispanic/Latino	130 (71.0)	219 (49.9)	**349 (56.1)**
Hispanic/Latino	39 (21.3)	98 (22.3)	**137 (22.0)**
Other/Unknown	14 (7.7)	122 (27.8)	**136 (21.9)**
**COVID-19 vaccination status^¶^**			
Received a booster	57 (31.1)	114 (26.0)	**171 (27.5)**
Fully vaccinated	85 (46.4)	154 (35.1)	**239 (38.4)**
<5 months before index date	12 (6.6)	28 (6.4)	**40 (6.4)**
≥5 months before index date	70 (38.3)	88 (20.0)	**158 (25.4)**
Timing of vaccination unknown	3 (1.6)	38 (8.7)	**41 (6.6)**
Partially vaccinated	2 (1.1)	15 (3.4)	**17 (2.7)**
Not vaccinated	36 (19.7)	129 (29.4)	**165 (26.5)**
Unknown	3 (1.6)	27 (6.2)	**30 (4.8)**
**Previous COVID-19 infection status**			
Previous infection	11 (6.0)	22 (5.0)	**33 (5.3)**
No previous infection	170 (92.9)	306 (69.7)	**476 (76.5)**
Unknown	2 (1.1)	111 (25.3)	**113 (18.2)**
COVID-19 case status**			
Confirmed	172 (94.0)	178 (40.5)	**350 (56.3)**
Probable	11 (6.0)	49 (11.2)	**60 (9.6)**
Not a case	0 (—)	204 (46.5)	**204 (32.8)**
Unknown	0 (—)	8 (1.8)	**8 (1.3)**

* Persons self-reported their race (White, Black, Asian, American Indian or Alaska Native, or Native Hawaiian or other Pacific Islander), ethnicity (Hispanic/Latino or non-Hispanic/Latino), and gender (male or female) from lists of options and had the opportunity to state another option if their race, ethnicity, or gender was not listed.

^†^ Age at index date was determined from date of birth or self-reported age.

^§^ The “other/multiple” race category included American Indian or Alaska Native, Native Hawaiian or other Pacific Islander, another race specified by the person not in the provided list, or multiple races.

^¶^ Received a booster dose was defined as having received an additional dose after completion of the primary COVID-19 vaccination series before the index date. Fully vaccinated was defined as completion of the primary vaccination series ≥2 weeks before the index date and stratified into completion <5 months or ≥5 months before the index date. Some persons who were fully vaccinated had unknown dates for completion of their primary vaccination series. Partially vaccinated was defined as having only 1 dose of a 2-dose series or completing the primary vaccination series <2 weeks before the index date.

** An index patient with a confirmed COVID-19 case was the first person with a positive SARS-CoV-2 nucleic acid amplification test result or antigen test result (through local or home testing) reported in a household. An index patient with a probable COVID-19 case was the first person with onset of any symptom consistent with COVID-19, but without a positive SARS-CoV-2 test confirmation, reported in a household. A confirmed case in a household contact was receipt of a positive SARS-CoV-2 nucleic acid amplification test result or antigen test result (through local or home testing) reported ≤14 days after the index date. A probable case in a household contact was the presence of any symptom consistent with COVID-19 during the same 14-day period but without a positive SARS-CoV-2 test confirmation.

Index dates occurred during November 21, 2021–February 3, 2022.^¶¶¶^ Among index patients, 172 (94.0%) had a positive SARS-CoV-2 test result (confirmed COVID-19) and 11 (6.0%) had COVID-19–compatible symptoms but without SARS-CoV-2 test confirmation (probable COVID-19). Among 439 household contacts, cases were identified in 227 (51.7%), including 178 (40.5%) confirmed and 49 (11.2%) probable cases; among the remaining household contacts, 204 (46.5%) were classified as non–COVID-19 patients and eight (1.8%) as having unknown status.**** A negative SARS-CoV-2 test result was reported on the day of or after symptom onset by 38.8% (19 of 49) of household contacts classified as having probable COVID-19 and 68.6% (140 of 204) of those classified as not having COVID-19. The median interval between index patient onset date and household contact onset date was 4 days (IQR = 2–7 days) (Figure 1).

**FIGURE 1:**
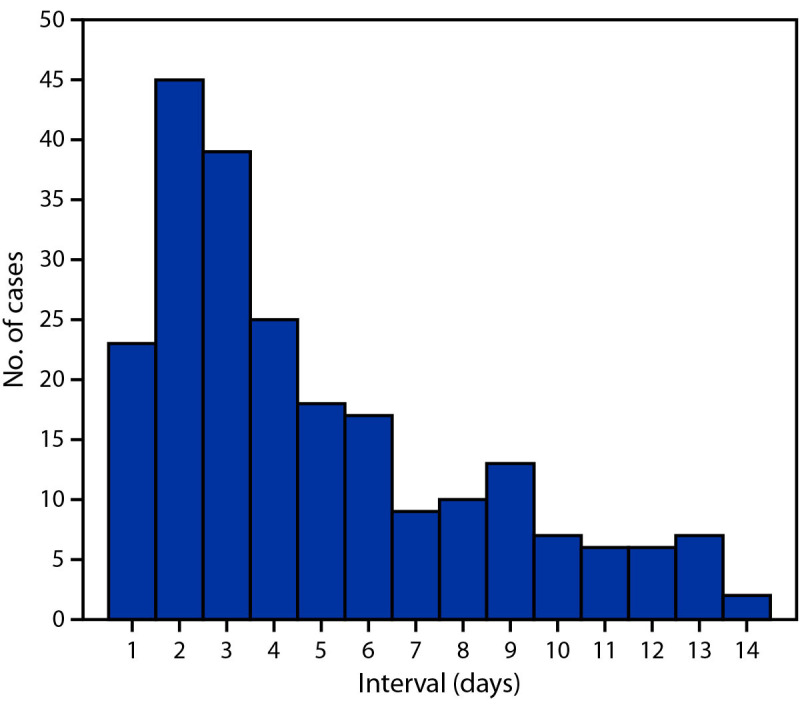
Interval*^,†^ between index patient onset date and household contact onset date — four U.S. jurisdictions, November 2021– February 2022 * The interval was estimated by calculating the number of days between the symptom onset or positive test result date for the index patient and that of the household contact. For both index patients and household contacts, the onset date was either the date of SARS-CoV-2 positive test result or date of symptom onset, whichever occurred first. ^†^ Transmission can occur within a household setting on the first day an index patient is infected or on any subsequent day during which they are still shedding viable virus.

Most index patients (88.4%, 152 of 172) and household contacts (78.7%, 140 of 178) with confirmed cases reported COVID-19–compatible symptoms. Of those with known SARS-CoV-2 infection history, eleven (6.1%) of 181 index patients and nine (4.7%) of 192 household contacts with confirmed or probable COVID-19 reported a previous SARS-CoV-2 infection.

Transmission occurred within 67.8% (124 of 183) of households, and the overall AR among household contacts with known status was 52.7% (227 of 431) (Figure 2). Similar ARs were observed across age groups for household contacts, including those aged 0–4 years (51.2%, 21 of 41). ARs were high across all household contact vaccination categories but lowest among those who received a booster dose (47.8%, 54 of 113) or were fully vaccinated <5 months before the index date (50.0%, 14 of 28). The AR among household contacts with previous SARS-CoV-2 infection was 40.9% (9 of 22) compared with 59.8% (183 of 306) among those without previous infection (p-value = 0.08).

**FIGURE 2:**
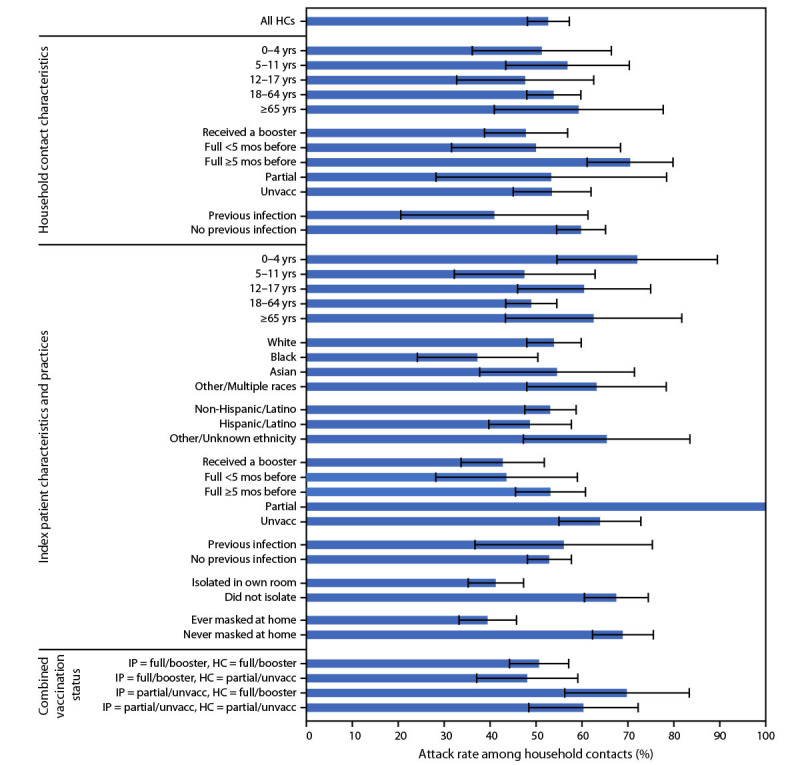
SARS-CoV-2 infection attack rates* among household contacts (N = 431) with known case status, by household contact characteristics,^†^^,^^§^ index patient characteristics and practices,^†^^,^^§^^,^^¶^ and combined vaccination status** — four U.S. jurisdictions, November 2021–February 2022 **Abbreviations:** Full = fully vaccinated; HC = household contact; IP = index patient; Partial = partially vaccinated; Unvacc = unvaccinated. * Analysis of attack rates among HCs excluded persons with unknown case status or “unknown” categorization within a given stratum. 95% CIs for attack rates are represented by error bars. ^†^ Age at index date was determined from date of birth or self-reported age. ^§^ Received a booster dose was defined as having received an additional dose after completion of the primary COVID-19 vaccination series before the index date. Fully vaccinated was defined as completion of the primary vaccination series ≥2 weeks before the index date and stratified into completion <5 months or ≥5 months before the index date. Some persons who were fully vaccinated had unknown dates for completion of their primary vaccination series. Partially vaccinated was defined as having only 1 dose of a 2-dose series or completing the primary vaccination series <2 weeks before the index date. ^¶^ Persons reported their race (White, Black, Asian, American Indian or Alaska Native, or Native Hawaiian or other Pacific Islander) and ethnicity (Hispanic/Latino or non-Hispanic/Latino) from lists of options and had the opportunity to state another option if their race or ethnicity was not listed. The “other/multiple races” category included American Indian or Alaskan Native, Native Hawaiian or other Pacific Islander, another race specified by the person not in the provided list, or multiple races. ** Analysis for attack rates by combined vaccination status combined persons who were fully vaccinated or had received a booster dose into one category (full/booster) and persons who were partially vaccinated or unvaccinated into another category (partial/unvacc).

Household contact ARs ranged from a low of 47.5% (19 of 40) when the index patient was aged 5–11 years to a high of 72.0% (18 of 25) when the index patient was aged 0–4 years. The ARs among household contacts by index patient vaccination status were lowest among those who received a booster dose (42.7%, 47 of 110) and those who were fully vaccinated <5 months before the index date (43.6%, 17 of 39). The AR was lower among household contacts of index patients who isolated (41.2%, 99 of 240) compared with those of index patients who did not isolate (67.5%, 112 of 166, p-value<0.01). The AR was lower among household contacts of index patients who reported ever wearing a mask at home during their potentially infectious period (39.5%, 88 of 223) compared with household contacts of index patients who reported never wearing a mask at home during this period (68.9%, 124 of 180, p-value<0.01). Subanalyses focusing on secondary household transmission demonstrated a similar interval (median = 3 days, IQR = 2–5) (Supplementary Figure 1, https://stacks.cdc.gov/view/cdc/114723) and similar patterns in ARs (Supplementary Figure 2, https://stacks.cdc.gov/view/cdc/114722).

## Discussion

Omicron infection resulted in high ARs among household contacts in this investigation, particularly among those who lived with index patients who were not vaccinated or who did not practice prevention measures (isolating or ever wearing a mask at home). The estimated overall AR in this investigation is consistent with the range of ARs observed in other Omicron transmission studies^††††^ (*3*), and higher than those associated with some other SARS-CoV-2 variants.^§§§§^ These findings underscore the importance of implementation of multicomponent prevention measures for reducing SARS-CoV-2 transmission in household settings, including from the Omicron variant (*4*).

ARs were consistently high across household contact and index patient age groups, including those aged 0–4 years. This age group is currently not eligible for vaccination and is a population in which some prevention strategies, such as isolation and mask use, might be difficult or impractical to implement. These findings further highlight young children's potential contribution to household transmission of SARS-CoV-2, as well as their ongoing susceptibility to infection when SARS-CoV-2 is introduced in the home^¶¶¶¶^ (*5*).

These findings are subject to at least six limitations. First, this investigation used a convenience sample of persons with sequence-confirmed Omicron infections, and participation in this investigation was voluntary. The small sample size, especially for certain stratum-specific ARs, may limit overall generalizability of the results. Households with high transmission or with more attention to public health measures may have been more likely to participate. Second, the investigation relied primarily on self-reported data. Vaccination status was not always verified, and the analysis did not account for potential variations in prevention practices (e.g., frequency of mask use). Third, COVID-19 prevention measures (vaccination, isolation, and mask use) are likely highly correlated within households, and the identified risk factors might not be independent predictors of transmission. Fourth, the interval analysis reflected time between dates of a positive test result or symptom onset, not date of infection, and did not account for duration of symptoms and prevention strategies, such as frequency of mask use. Fifth, this investigation did not definitively distinguish between secondary and potential tertiary cases within a household. Finally, this investigation occurred during a period when testing and sequencing capacity was strained and when many persons traveled and attended gatherings, increasing the possibility that household contacts had unknown SARS-CoV-2 exposures outside the home (*6*). Because SARS-CoV-2 testing was not available for all household contacts, ability to detect asymptomatic infections was limited. Without sequencing results for all household contact cases, it was not possible to confirm that transmission occurred from index patients to household contacts or that household contacts were infected with the same variant.

 The findings from this investigation reinforce the importance of multi-component prevention strategies, including up-to-date vaccination, isolation of infected persons, and mask use at home, to reduce Omicron transmission in household settings.

**Summary**

**What is already known about this topic?**

The SARS-CoV-2 B.1.1.529 (Omicron) variant contributed to a surge of SARS-CoV-2 infections in the United States during December 2021–January 2022.

**What is added by this report?**

In a study of household transmission in four U.S. jurisdictions, Omicron infection resulted in high transmission among household contacts, particularly among those who lived with index patients who were not vaccinated or who did not take measures to reduce the risk of transmission to household contacts.

**What are the implications for public health practice?**

Multicomponent COVID-19 prevention strategies, including up-to-date vaccination, isolation of infected persons, and mask use at home, are important to reduce Omicron transmission in household settings.

## Footnotes

* These authors contributed equally to this report.

† In this investigation, a confirmed case in a household contact was defined as having received a positive SARS-CoV-2 nucleic acid amplification test result or antigen test result ≤14 days after the index date (date of the index patient’s symptom onset or positive SARS-CoV-2 nucleic acid amplification test result or antigen test result), and a probable case in a household contact was defined as the presence of COVID-19–compatible symptoms during the same 14-day period but without a positive SARS-CoV-2 test confirmation. Persons without symptoms and who did not have a positive SARS-CoV-2 test result were not considered to have a case of COVID-19. Analysis of AR among household contacts excluded eight persons with unknown case status (persons for whom it was not known whether COVID-19–compatible symptoms were present and whether SARS-CoV-2 testing had occurred [or if testing occurred, the results were unknown]).

§ Jurisdictions identified persons who were considered potentially eligible for participation through obtaining laboratory line lists of persons who had sequence-confirmed Omicron (B.1.1.529 lineage and its sublineages) or whose sequencing results were pending. Two jurisdictions attempted to contact all households on their line lists, and two jurisdictions attempted to contact households on their line lists based on specimen collection date.

¶ Persons were provided with the following list of signs and symptoms compatible with COVID-19: fever or chills, cough, shortness of breath or difficulty breathing, fatigue, muscle or body aches, headache, new loss of taste or smell, sore throat, congestion or runny nose, nausea or vomiting, and diarrhea or abdominal pain during the course of their recent illness. Persons who reported any signs or symptoms during the course of their recent illness were considered to have COVID-19–compatible symptoms. Persons who only had signs or symptoms (and no positive SARS-CoV-2 test result) were considered to have a probable case.

** Persons provided retrospective information about any SARS-CoV-2 testing that they chose and were able to perform. Thus, whether someone was tested and how many times they were tested depended on individual and social factors. Interviewers encouraged household contacts who had not received testing to receive testing if the telephone interview occurred ≤14 days after the index date and instructed them to call back with test results. When possible, SARS-CoV-2 testing data were supplemented with or verified using state or jurisdiction registry data.

†† Persons with probable cases included symptomatic persons who did not have SARS-CoV-2 testing and symptomatic persons who received negative SARS-CoV-2 test results.

§§ When possible, vaccination data were supplemented with or verified using state or jurisdiction registry data.

¶¶ Received a booster dose was defined as having received an additional dose after completion of the primary COVID-19 vaccination series before the index date. Fully vaccinated was defined as completion of the primary vaccination series ≥2 weeks before the index date and stratified into completion <5 months or ≥5 months before the index date. Some persons who were fully vaccinated had unknown dates for completion of their primary vaccination series. Partially vaccinated was defined as having only 1 dose of a 2-dose series or completing the primary vaccination series <2 weeks before the index date.

*** Seven days was chosen for this analysis, because 75% of cases occurred ≤7 days after the index date.

††† 45 C.F.R. part 46.102(l)(2), 21 C.F.R. part 56; 42 U.S.C. Sect. 241(d); 5 U.S.C. Sect. 552a; 44 U.S.C. Sect. 3501 et seq.

§§§ Among the 204 excluded households, 143 did not meet eligibility criteria, 47 were excluded because the index patient could not be identified, 11 were excluded because household contacts had confirmed or probable cases and were known to have a SARS-CoV-2 exposure other than exposure to the index patient, and three were excluded when sequencing results (pending at the time of interview) indicated a variant that was not Omicron. All but one of the 183 included households had sequence-confirmed Omicron; this one household had probable Omicron through variant specific qPCR in which the specimen had mutations consistent with the Omicron variant (K417N+ and L452R−).

¶¶¶ The median interval between index date and date of interview was 24 days (IQR = 17–29 days).

**** Case status of household contacts was unknown if the occurrence of COVID-19–compatible symptoms was not known and if the contact’s testing results were unknown.

††††
https://www.medrxiv.org/content/10.1101/2021.12.27.21268278v1

§§§§
https://www.medrxiv.org/content/10.1101/2022.01.09.22268984v1

¶¶¶¶
https://www.medrxiv.org/content/10.1101/2021.08.16.21262121v2
